# Essential newborn care practice and its predictors among mother who delivered within the past six months in Chencha District, Southern Ethiopia, 2017

**DOI:** 10.1371/journal.pone.0208984

**Published:** 2018-12-11

**Authors:** Abera Mersha, Nega Assefa, Kedir Teji, Shitaye Shibiru, Rasha Darghawth, Agegnehu Bante

**Affiliations:** 1 Department of Nursing, College of Medicine and Health Sciences, Arba Minch University, Arba Minch, Ethiopia; 2 School of Public Health, College of Health and Medical Sciences, Haramaya University, Harar, Ethiopia; 3 School of Nursing and Midwifery, College of Health and Medical Sciences, Haramaya University, Harar, Ethiopia; 4 Business Development Officer with CARE Ethiopia and Cuso International, Monitoring and Evaluation Advisor, Harar, Ethiopia; ESIC Medical College & PGIMSR, INDIA

## Abstract

**Introduction:**

Components of essential newborn care and neonatal resuscitation are proven interventions for reducing neonatal mortality rate and stillbirth rates. Various studies have been conducted, but they failed in assessing health workers that delivered essential newborn care, facets of the health care system, and different traditional beliefs. As such, the primary aim of this study is to fill the gaps of the aforementioned previous studies, assess mothers’ current practice of essential newborn care and identify factors affecting newborn care practices in Chencha District, Southern Ethiopia.

**Methods:**

A mixed type, community-based cross sectional study was conducted among 630 study participants by using one-stage cluster sampling method. Three focus group discussions (FGD) with purposively selected 18 mothers were involved for qualitative study. Data entry was carried out by Epi data version 3.1 and analysis was done by SPSS window version 22. Binary logistic regression was used to identify predictors. Qualitative data were analyzed deductively by using thematic framework analysis approach by using Open Code version 4.02.

**Results:**

This study found that 38.4% of mothers had good practices in essential newborn care. Of the neonates, 52.9% received safe cord care, 71.0% received optimal thermal care and 74.8% had good neonatal feeding. Factors such as mothers receiving antenatal care, attending pregnant mothers meetings, receiving immediate postnatal care, wealth index, whether a complication was faced during delivery and overall knowledge of mothers were statistically significantly associated with practice.

**Conclusions:**

This study indicated that the current rate of essential newborn care practice was low. As such, strengthening the provision of antenatal and postnatal care services, information communication education and behavioral change communications on essential newborn care are recommended.

## Introduction

Globally, there have been great strides made in reducing the number of neonatal deaths, declining from 5.1 million in 1990 to 2.7 million in 2015. However, the decline in neonatal mortality from 1990 to 2015 has been far slower than that of post-neonatal under-5 mortality. This pattern applies to most low- and middle-income countries, as current global data shows that approximately 99% of maternal and newborn mortality occurs in the developing world [1, 2]. Annually, approximately 1.16 million African babies die in the first 28 days of life. The burden of neonatal mortality and ill health is concentrated among the marginalized and poorest populations in countries of Sub-Saharan Africa and South Asia. The majority of neonatal mortality in developing countries is related to conditions of labor, intrapartum and poor immediate newborn care practices [3–5]. More than 60% of infant and 40% of under-five deaths in Ethiopia are neonatal deaths. This dire situation calls for extensive health care services [6].

To illustrate the, UNICEF has stated that, “the period around birth constitutes a critical window of opportunity for prevention and management of maternal and newborn complications, which can otherwise prove fatal” [7]. Essential newborn care (ENC) is a set of measures every newborn baby requires regardless of where it is born or its size. It is designed to protect the newborn in adverse environmental condition [1, 8, 9] and is a framework that should be applied immediately after birth, continued at least for the first seven days. Components of ENC and neonatal resuscitation are proven interventions for reducing neonatal mortality rate and stillbirth rate [10].

There are inequities affecting the access and utilization of maternal, neonatal and child health services due to women’s age, education, household wealth index and distance of the women’s household from the nearest health facility still exist in rural Ethiopia [11]. Different studies conducted in Ethiopia stated that the prevalence of essential newborn care practice was low [12–14].

Community-based newborn care implementation programs in Ethiopia involve the scaling-up of community-based maternal and newborn health services, including the provision of immediate newborn care, initial stimulation and resuscitation of the newborn baby, prevention and management of hypothermia, management of pre-term and low birth weight (LBW) neonates, and management of neonatal sepsis and very severe disease at community level [15].

However, various studies conducted in Ethiopia have failed in assessing the health workers that implemented ENC, health care system; there difference in socio-demographic characteristics and cultural background. To the author’s knowledge, studies have also neglected to assess the proportion of children who received ENC and factors that hinders the provision of the services among mothers. As such, the purpose of this study was to fill those gaps, assessing mother’s recent status of essential newborn care practice and identify factors hindering and promoting these practices in Chencha District, Southern Ethiopia.

## Methods

### Study setting, period and design

This community-based mixed study was conducted in Chencha District between February 8 and 28^th^, 2017. Chencha District is one of the Districts in Gamo Gofa Zone within the South Nations, Nationalities and Peoples’ Region (SNNPR) of Ethiopia and it is located 562 Km Southwest of Addis Ababa, the capital city of Ethiopia. Chencha District has 50 kebeles (five urban and 45 rural), towns in Chencha district include Chencha, Dorze, Dokko and Ezo. The district has a total population of 140,183 people (68,970 males and 71,213 females), approximately 28,609 households [16].

### Population

All mothers who gave birth in six months prior to the study period constituted the source population. Mothers who were found in selected clusters comprised the study population for this study. The sampling unit was households and the study unit was mothers [17].

### Eligibility criteria

The inclusion criteria were all mothers who gave birth in the past six months prior to the study period and were residents for at least 6 months in the study area was included in this study. Whereas, the exclusion criteria were mothers who were not mentally competent, seriously ill and whose delivered baby died prior to the data collection period [17].

### Sample size determination

Epi info7 software Stat Cal was used to calculate the sample sizes. To determine the prevalence of essential newborn care practice, a single population proportion was used and to identify predictors, a double population proportion was used. The final sample size was derived through adding non-response rate of 10% to the larger sample size which was 561. So, the calculated sample size for quantitative study was 618. However, due to the cluster effect the final sample used for this study was 630 [17]. The design effect of 1.5 was used to calculate the sample sizes. For the qualitative part of the study, three focus group discussions (FGD) with six mothers on each group were involved depending on idea saturation.

### Sampling procedure

**Fig 1:**
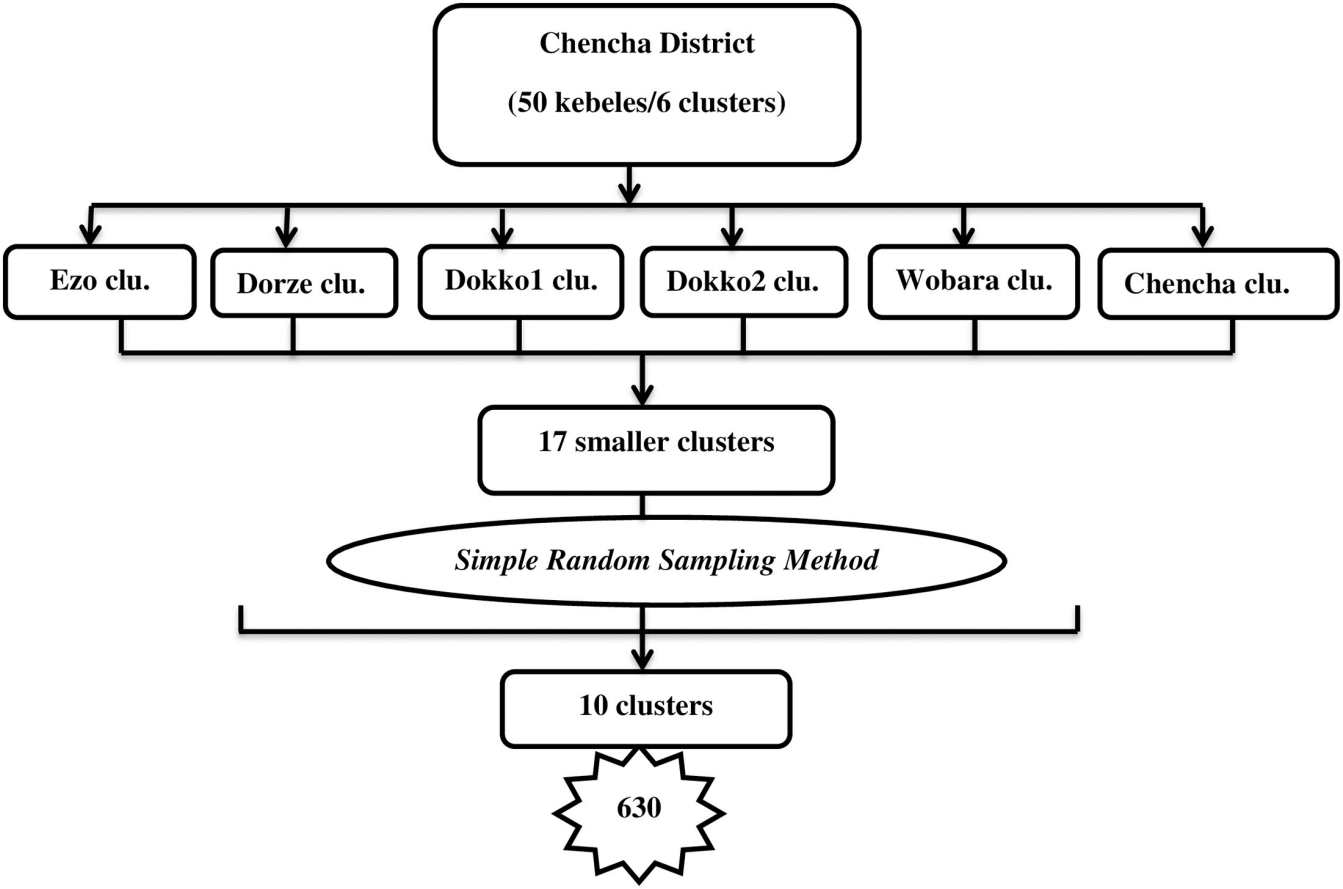
Schematic presentation of sampling procedure for the study conducted among mothers who delivered in the past six months in Chencha District, Southern Ethiopia, 2017.

For quantitative data, the one-stage cluster sampling method was used. For the qualitative data, purposive sampling was used. The Chencha district has fifty kebeles or six clusters based on topography and catchment areas, relative to the health centers. Initially, the 6 clusters were clustered in to seventeen smaller clusters. From the seventeen clusters, nine clusters were selected by simple random sampling method. However, to reach the calculated sample size, one additional cluster was added again by simple random sampling from the residual clusters. As such, the final clusters selected for this study were 10 (Fig 1).

### Data collection methods

A pre-tested, structured interview was administered and used to collect quantitative data. An interview guide for discussion was used to collect qualitative information. The data were collected by trained 7 diploma nurses and supervised by two BSc holder nurses who were fluent in the local language, *Gammogna*. The data collectors collected the information through a face to face interview of mothers at the household level. To generate qualitative information, an FGD was conducted. Each session of the FGD was tape recorded after getting written and signed voluntary consent from the FGD participants. Those participants were selected by principal investigator and the discussion was conducted until a saturation of ideas occurred within the group. The discussion was moderated by principal investigator and one other assistant (data collector) took notes and recorded all the information of the FGD.

### Study variables and measurements

Essential newborn care practice was the dependent variable and socio-demographic and socio-economic characteristics, maternal and child health services, knowledge about ENC, health care system and traditional beliefs were independent variables for this study. The detail descriptions and measurements are given below (Table 1).

**Table 1:** Description of variables and measurements for the study in Chencha District, Southern Ethiopia, 2017.

Variables	Description	Measurements/Categories
**Dependent/Outcome Variable**		
**Essential newborn care practice**	The practice was good for mothers who were practice three domains (safe cord care, optimal thermal care, and good neonatal feeding) appropriately whereas the practice was poor if one component was missed from three domains [13, 18].	Those mothers who practice good essential new born care were coded as ‘1’ and poor practice of essential new born care were coded as ‘0’.
**Composite Variables**		
**Safe cord care**	Defined as the use of a clean cutting instrument to cut the umbilical cord (boiled new, used blade or scissor) plus clean thread, cord tie or cord clamp and no any substance applied on the cord stump [13, 18].	Safe cord care was categorized as ‘Yes’ and ‘No’. Yes was labeled as ‘1’ and No was labeled as ‘0’.
**Optimal thermal care**	Means wiped off/ dried the baby within ten minute, wrapped in new or clean and dry old cloth and washing the body of the newborn by warm water after 24 hour of delivery to prevent hypothermia [18, 19].	Optimal thermal care was categorized as ‘Yes’ and ‘No’. Yes was labeled as ‘1’ and No was labeled as ‘0’.
**Good neonatal feeding**	Known as initiating breastfeeding within the first one hour after birth, giving no prelacteal and feeding the child with colostrum [18].	Good neonatal feeding was categorized as ‘Yes’ and ‘No’. Yes was labeled as ‘1’ and No was labeled as ‘0’.
**Independent Variables**		
**Knowledge about essential newborn care**	Knowledge was good for mothers who responded greater than 50% of knowledge related questions correctly whereas knowledge was poor for mothers who responded less than or equal to 50% of knowledge related questions [20].	Good knowledge about essential newborn care was coded as ‘1’ and poor knowledge was coded as ‘0’.
**Marital status**	Mothers current marital status	The response was categorized as: married = “1”, divorced = “2”, widowed = “3” and separated due to work = “4”
**Place of residence**	Actual place of residence in which the mother lives	Categorized in to two: “1” = Urban and “2” = Rural.
**Educational status**	Maternal level of education	Categorized in to 5 groups as, cannot able to read and write = “1”, can read and write = “2”, grade (1–8) = “3”, grade (9–12) = “4” and college and above = “5”.
**Occupation**	Current employment status and specific occupation of respondent mother	Categorized as: housewife = “1”, merchant = “2”, government employer = “3” and daily laborer = “4”.
**Parity**	Number of births a woman have including current birth	The responses was categorized in to three categories as: 1^st^ birth order = “1”, 2^nd^– 4th = “2” and ≥5^th^ birth order = “3”.
**Wealth index**	Using EDHS household assets questions and principal component analysis was done	Ranked in to three categories: 1^st^ quintile, 2^nd^ quintile and 3^rd^ quintile.
**ANC frequency**	Having health facility visit for pregnancy follow up by skilled or professional health care provider.	Categorized in to three categories: 1–3 visit = “1”, 4 visit = “2” and ≥5 visit = “3”
**Distance to the health care institutions**	Approximate distance (in km) to the nearest health care institutions which was reported by respondent	Categorized in to two: “1” = <5km and “2” = ≥5km

### Data quality control

In order to ensure quality, the questionnaire was initially drafted in English language, and then translated in to local language, *Gammogna* by verified translators. Lastly, before data collection, the questionnaire was back translated in to English to ensure accuracy. Questionnaires were pre-tested in an area with a population with a similar socio-demographic status. Data were checked for completeness, accuracy, clarity and consistency before being entered in to software. Proper coding and categorization of data were maintained for the quality of the data to be analyzed. Double data entry was used to ensure validity and compare to the original data.

### Data analysis and processing

The quantitative data were coded, cleaned, edited and entered into Epi data version 3.1, then exported to SPSS window version 22 for analysis. Wealth quintiles were determined by using a Principal Component Analysis (PCA). Binary logistic regression was used to assess the association between each independent variables and outcome variable. Hosmer-Lemeshow statistic and Omnibus tests conducted done for model fitness. All variables with P<0.2 in the bivariate analysis were included in the final model of multivariate analysis in order to control all possible confounders. In addition, variables which were significant in previous studies and from context point of view included in the final model even if the above criteria was not meet. A variance inflation factor >10 and standard error >2 were considered as suggesting the existence of multi co-linearity. The direction and strength of statistical association was measured by an odds ratio with 95% CI. Adjusted odds ratio along with 95%CI was estimated to identify predictors for essential newborn care practice. In this study P-value < 0.05 was considered to declare a result as a statistically significant association. The focus group discussions audios were initially transcribed verbatim in the local language, *Gammogna*, and then translated into English transcripts by the principal investigator. Data were analyzed deductively by using a thematic framework analysis approach and qualitative data analysis software Open Code version 4.02 was used. Each transcript was carefully screened and coded. Those codes were in turn grouped into major themes representing positive essential newborn care practices such as cord care, thermal care and neonatal feeding.

### Ethics approval and consent to participate

Ethical clearance was obtained from Haramaya University, College of Health and Medical Sciences, Institutional Health Research Ethics Review Committee (HU-IHRERC). All study participants were informed about the purpose of the study, their right to refuse participation and written and signed voluntary consent was obtained from all study participants prior to the interview. The respondents were also informed that the information obtained from them was treated with utmost confidentiality.

## Results

### Socio-demographic and economic characteristics

In this study, 630 participants responded to the questionnaire, with a total response rate of 100%. The majority of the respondents were in the age group 25–34 and the mean age of study participants were 29.62 (±5.082 SD). Of the respondents, the majority were married 584 (92.7%) and lived in rural locations constitutes 400 (63.5%). Orthodox Christianity was the dominant religion 489 (77.6%) among study participants (Table 2).

**Table 2:** Socio-demographic/economic characteristics of study participants in Chencha District, Southern Ethiopia, 2017(*n* = *630*).

Variables	Frequency	Percentage
**Age**		
15–24	94	14.9
25–34	420	66.7
35–44	116	18.4
**Marital status**		
Married	584	92.7
Divorced	9	1.4
Widowed	5	0.8
Separated due to work	32	5.1
**Religion**		
Orthodox	489	77.6
Protestant	141	22.4
**Educational status**		
Cannot able to read and write	208	33.0
Can read and write	76	12.1
Grade 1–8	183	29.0
Grade 9–12	99	15.7
College and above	64	10.2
**Occupation of mother**		
House Wife	420	66.7
Merchant	136	21.6
Government Employer	59	9.4
Daily Laborer	15	2.4
**Place of residence**		
Urban	230	36.5
Rural	400	63.5
**Wealth index in quintile**		
1^st^ quintile	224	35.6
2^nd^ quintile	198	31.4
3^rd^ quintile	208	33.0

This table is published with other objectives on American Journal of Nursing Science. Vol. 6, No. 5, 2017, pp. 426–432. doi: 10.11648/j.ajns.20170605.17

### Maternal and child health services

Of the respondents, 533 (84.6%) had ANC follow up. Two hundred and eleven (39.6%) had one to three visits, 279 (52.4%) had four visits and 43 (8.1%) had five and more visits. During ANC follow up the majority 522 (97.9%) were advised about ENC. Two hundred twenty three (35.4%) had delivered at health center and 421 (66.8%) had immediate postnatal care. The majority of the neonates 593 (94.1%) were immunized and 528 (83.8%) mothers did not face any complications during delivery. Five hundred thirty eight (85.4%) study participants gave birth through spontaneous vaginal delivery, 83 (13.2%) included at instrumental delivery and 9 (1.4%) had caesarean delivery as data has been published elsewhere [17] (Table 3). Of the respondents, 437 (69.4%) were assisted by skilled birth attendants and 68 (10.8%), 26 (4.1%), 43 (6.8%), 31 (4.9%) and 25 (4%) were assisted by a family member, neighbor, relatives/mother in law, traditional birth attendants and health extension workers during delivery.

**Table 3:** Maternal and child health services among study participants in Chencha District, Southern Ethiopia, 2017 *(n = 630)*.

Variables	Frequency	Percentage
**ANC**		
Yes	533	84.6
No	97	15.4
**Attended monthly pregnant mothers meeting**		
Yes	517	82.1
No	113	17.9
**Place of delivery**		
Health center	223	35.4
Hospital	214	34
Health post	25	4.0
Home	168	26.6
**Faced complication during delivery**		
Yes	102	16.2
No	528	83.8
**Care giver during postnatal period**		
HEW	284	45.1
Family/mother in law	293	46.5
Neighbor	14	2.2
Mother of the women	39	6.2
**Immediate postnatal care**		
Yes	421	66.8
No	209	33.2
**Party**		
1	79	12.5
2–4	501	79.5
≥5	50	7.9

This table is published with other objectives on American Journal of Nursing Science. Vol. 6, No. 5, 2017, pp. 426–432. doi: 10.11648/j.ajns.20170605.17

### Essential newborn care practice

Of the respondents, 251 (39.8%) applied substances on the stump, 176 (70.1%) applied ointment/powder, 70 (27.9%) applied butter, 4 (1.6%) applied animal dung and 1 (0.4%) applied ash on the stump. Eye care was given for 456 (72.4%) of the babies and 407 (89.3%) stated the care givers were health extension workers and 49 (10.7%) stated the care givers were health professionals (nurses and midwives). Colostrum was given for 568 (90.2%) of the babies of respondents after delivery and pre-lacteal feeding was given for 90 (14.3%) of the babies. Among those who gave pre-lacteal feeding 53 (58.9%) of the mothers gave plain water, 28 (31.1%) gave butter, 8 (8.9%) gave animal milk and 1 (1.1%) gave honey. Six hundred and six (96.2%) of the newborn babies had wiped and 601 (95.4%) had wrapped the baby in cloth after delivery (Table 4).

**Table 4:** Mothers practice on essential newborn care in Chencha District, Southern Ethiopia, 2017(*n* = *630*).

Variables	Frequency	Percentage
**Instrument boiled**		
Yes	554	87.9
No	76	12.1
**Applied substances on the stump**		
Yes	251	39.8
No	379	60.2
**Cord tie**		
String/thread	184	29.2
Cord tie	441	70.0
Cord clamp	5	0.8
**Temperature of water used to bath**		
Warm	597	94.8
Cold	33	5.2
**Wiped within ten minute after delivery**		
Yes	606	96.2
No	24	3.8
**Wrapped by clothe after delivery**		
Yes	601	95.4
No	29	4.6
**Type of cloth used to wrap**		
New cloth	293	48.7
Clean and dry old cloth	289	48.1
Soiled and old cloth	19	3.2
**Gave colostrum**		
Yes	568	90.2
No	62	9.8
**Pre-lacteal feeding**		
Yes	90	14.3
No	540	85.7

**Fig 2:**
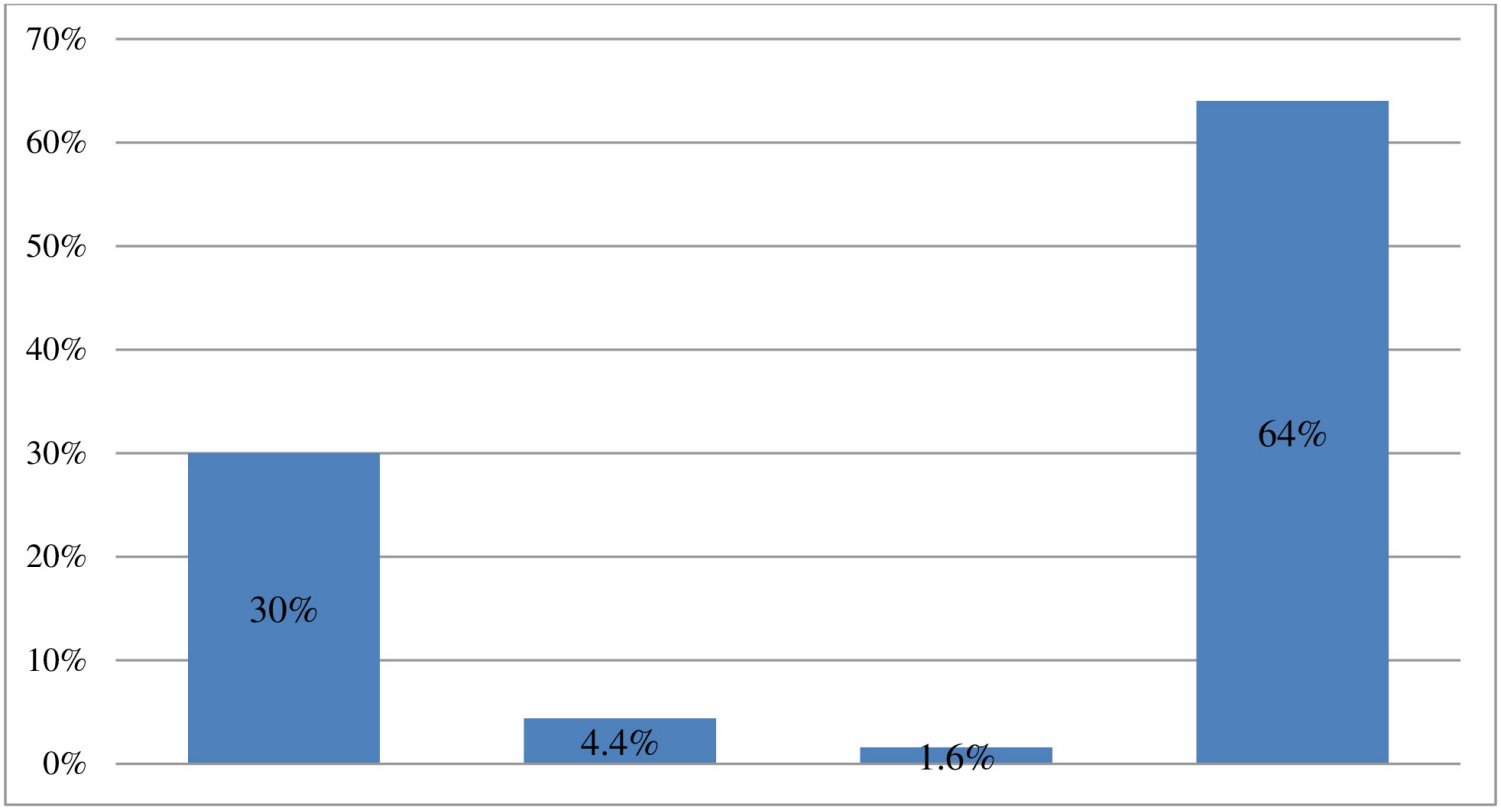
Instruments used by study participants to cut the cord in Chencha District, Southern Ethiopia, 2017(n = 630). Various instruments were used to cut the cord, with 189 (30%) respondents stating they used a new blade, 28 (4.4%) stating they used blade, and 10 (1.6%) stated using a knife. The majority, at 403 (64%) used a pair of scissors.

Various instruments were used to cut the cord, with 189 (30%) respondents stating they used a new blade, 28 (4.4%) stating they used blade, and 10 (1.6%) stated using a knife. The majority, at 403 (64%) used a pair of scissors (Fig 2).

The majority of the discussants in the qualitative findings stated that the cord was tied by thread or string and cut by a new blade if delivery occurs at home. Previously the cord was also tied by apart from the false banana plant *(zaniza)*. However, the communities noted they are moving away from this trend as improved awareness of the risks has been created by health extension workers. The other common practice reported by discussants was the application of fresh butter on and around the stump, whether delivery occurs at home or health care institutions.

*A 32 year-old FGD discussant noted that, “I delivered my newborn three months back at home by the help of traditional birth attendants. After delivery the attendants tied the cord by thread and cut it with a new blade that I bought from market. To cut the cord, attendants measure from the babies’ abdomen by their fingers. If the cord was tied and cut after 2 fingers from the abdomen of newborn it can dropped within 2 days and if tied and cut after 3 finger from the abdomen of newborn it can dropped within 3 days……. During the first 7 days after delivery I applied fresh butter on the stump to prevent dryness and to make it soft*.…”

**Fig 3:**
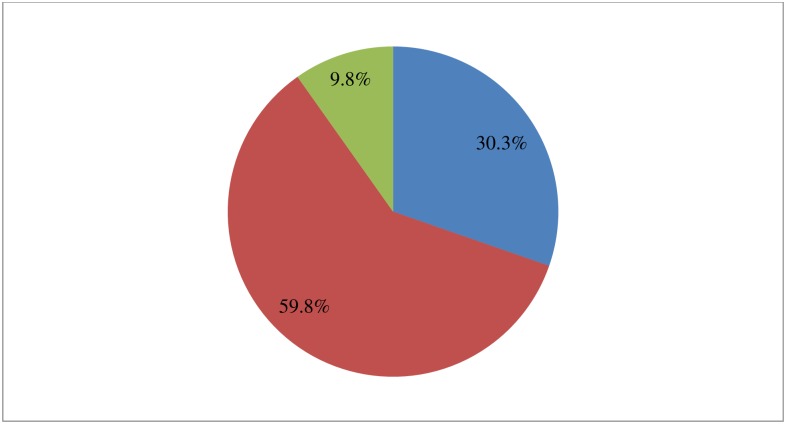
Breastfeeding initiated time among mothers in Chencha District, Southern Ethiopia, 2017(n = 630). Three hundred seventy seven (59.8%) of the study participants stated breastfeeding was initiated within one hour of delivery, 191 (30.3%) initiated immediately after delivery and 62 (9.8%) initiated after one hour of delivery.

Three hundred seventy seven (59.8%) of the study participants stated breastfeeding was initiated within one hour of delivery, 191 (30.3%) initiated immediately after delivery and 62 (9.8%) initiated after one hour of delivery (Fig 3).

*A 25-year old FGD discussant mother said that, “I had antenatal care follow up at health center every month. But, I delivered at home by the help of traditional birth attendants. After delivery the attendants gave expressed water from the “utha shedo” (false banana plant) before starting breastfeeding, they consider this important for newborns to initiate breast feeding. Then the attendants ordered me to wait for some time to breastfeed the newborn. In addition, I gave fluid from “mudha” (traditional herbs) for the newborn in order to facilitate the feeding and to prevent gastric disturbance*.

**Fig 4:**
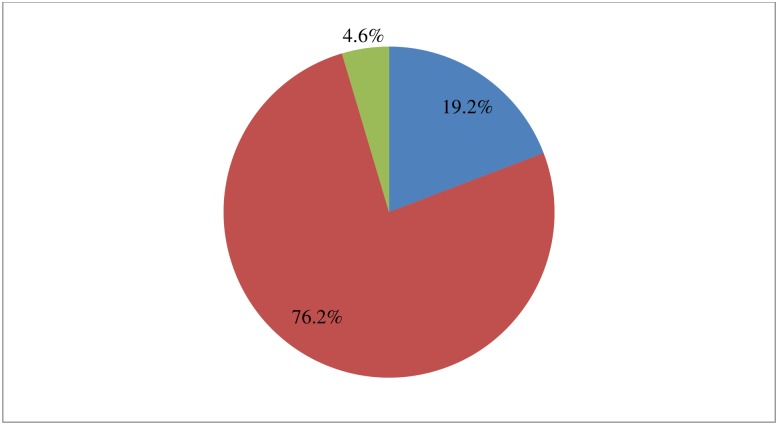
Time of bathing among mothers in Chencha District, Southern Ethiopia, 2017 (n = 630). Four hundred eighty (76.2%) of the study participants stated that the newborn had been bathed after 24 hours post-delivery, 121 (19.2%) had bathed their newborns immediate after delivery and 29 (4.6%) had bathed them before 24 hours post-delivery.

Four hundred eighty (76.2%) of the study participants stated that the newborn had been bathed after 24 hours post-delivery, 121 (19.2%) had bathed their newborns immediate after delivery and 29 (4.6%) had bathed them before 24 hours post-delivery (Fig 4).

The majority of the discussants in the qualitative findings stated that their newborns were washed in warm water because they consider as dirty or contaminated by blood and wrapped by piece of cloth (towel) or by a new cloth to prevent from cold immediate after delivery. There were also traditional practices like applying fresh butter on the head of a newborn, covering them in a cabbage leaf and then put hat on head or wrapped by a piece of cloth.

*A 27-year old FGD discussant said that, “I gave birth at home with the help of neighbors. Immediately after delivery, both mother and newborn were washed by warm water. The newborn was then wrapped in new cloth to protect him from the cold immediately after delivery, and both the mother and newborn lay close to fire. In our community, all mothers who have delivered live in a very hot house, heated by fire wood. The other important thing is to keep the newborn from cold fresh butter was putted on the head and covered by plant leaf, then cap was done or a small cloth was wrapped around the head*.”

In this study 38.4% of mothers had good practice on ENC. Of the neonates, 52.9% received safe cord care, 71.0% received optimal thermal care and 74.8% had good neonatal feeding (Table 5).

**Table 5:** Composite measure of essential newborn care practice in Chencha District, Southern Ethiopia, 2017(*n* = *630*).

Variables	Frequency	Percentage
**Safe cord care**		
Yes	333	52.9
No	297	47.1
**Optimal thermal care**		
Yes	447	71.0
No	182	28.9
**Good neonatal feeding**		
Yes	471	74.8
No	159	25.2

### Health care system

Out of the respondents, 176 (27.9%) stated that hospitals were available nearby, 124 (19.7%) had access to health center and 330 (52.4%) had access to health posts. Six hundred thirteen (97.3%) stated that these health care institutions create awareness about essential newborn care. A number of healthcare providers were involved in creating awareness including 495 (78.6%) health extension workers and health development army’s, 100 nurses (15.9%), 33 midwives (5.2%) and 2 public health officers (0.3%). Out of the study participants 572 (90.8%) stated that the distance to health care institutions were less than 5 km. The majority of the FGD discussants stated that health extension workers in home-based sessions create awareness about when to bath the newborn, when to initiate breastfeeding, cord care after delivery and about immunization. Before the prevalence health extension workers, the majority of mothers partook in traditional practices which can predispose the newborn to complications.

### Predictors for essential newborn care practice

In the multivariate model antenatal care, attending pregnant mothers meeting, it was found that immediate postnatal care, wealth index of 2^nd^ quintile, and a good degree of knowledge by the mother about ENC were positively associated with good ENC practice. However, facing a complication during delivery was negatively associated with good practice of ENC.

Mothers who had sought antenatal care were 3.13 times and who had attending pregnant mothers meeting were 2.90 times more likely to practice good essential newborn care, (AOR = 3.13, 95%CI: 1.47, 6.64) and (AOR = 2.90, 95%CI: 1.45, 5.82). The odds of good ENC practice were 3.27 among mothers who had immediate postnatal care and 7.36 among mothers who had good knowledge about ENC, (AOR = 3.27, 95%CI: 1.99, 5.35) and (AOR = 7.36, 95%CI: 2.77, 19.59). Mothers who had faced complication during delivery were 80% less likely to practice ENC (AOR = 0.20, 95%CI: 0.11, 0.37). Mothers with a wealth index in the 2^nd^ quintile were 74% more likely (AOR = 1.74, 95%CI: 1.12, 2.72) to practice good ENC (Table 6).

**Table 6:** Predictors for essential newborn care practice in Chencha District, Southern Ethiopia, 2017(*n* = *630*).

Variables	Essential newborn care practice		(95% CI)	
	Poor	Good	Crude OR	Adjusted OR
**Age**				
15–24	64(16.5%)	30(12.4%)	1	1
25–34	252(64.9%)	168(69.4%)	1.42(0.88–2.29)	0.89(0.49–1.58)
35–44	72(18.6%)	44(18.2%)	1.30(0.74–2.31)	0.98(0.48–1.98)
**Educational Status**				
Not read and write	140(36.1%)	68(28.1%)	1	1
Read and write	36(9.3%)	40(16.5%)	2.29(1.34–3.91)	1.41(0.75–2.65)
Elementary and above	212(54.6%)	134(55.4%)	1.30(0.91–1.87)	1.26(0.81–1.96)
**Occupation of mother**				
Employed	32(8.2%)	27(11.2%)	1.39(0.82–2.39)	0.81(0.43–1.52)
Unemployed	356(91.8%)	215(88.8%)	1	1
**Wealth index in quintile**				
1^st^ quintile	127(32.7%)	97(40.1%)	1	1
2^nd^ quintile	95(24.5%)	103(42.6%)	1.42(0.97–2.08)	1.74(1.12–2.72)*
3^rd^ quintile	166(42.8%)	42(17.4%)	0.33(0.22–0.51)	0.59(0.35–1.01)
**ANC**				
Yes	303(78.1%)	230(95.0%)	5.38(2.87–10.08)	3.13(1.47–6.64)**
No	85(21.9%)	12(5.0%)	1	1
**Attending pregnant mothers meeting**				
Yes	288(74.2%)	229(94.6%)	6.12(3.35–11.18)	2.90(1.45–5.82)**
No	100(25.8%)	13(5.4%)	1	1
**Skilled birth attendant**				
Yes	245(63.1%)	192(79.3%)	2.24(1.54–3.26)	0.87(0.53–1.42)
No	143(36.9%)	50(20.7%)	1	1
**Immediate PNC**				
Yes	212(54.6%)	209(86.4%)	5.29(3.46–7.98)	3.27(1.99–5.35)***
No	176(45.4%)	33(13.6%)	1	1
**Faced complication during delivery**				
Yes	85(21.9%)	17(7.0%)	0.27(0.16–0.47)	0.20(0.11–0.37)***
No	303(78.1%)	225(93.0%)	1	1
**Knowledge of mother**				
Good	307(79.1%)	237(97.9%)	12.51(4.98–31.35)	7.36(2.77–19.59)***
Poor	81(20.9%)	5(2.1%)	1	1

*Significant with P = 0.014,

**Significant with P = 0.003 and

***Significant with P<0.001

## Discussion

This study found that mothers’ good practice of ENC was 38.4%, 52.9% received safe cord care, 71% received optimal thermal care and 74.8% %) had good neonatal feeding. Mothers who had antenatal care were 13% and who had attending pregnant mothers meeting were 90% more likely to practice good ENC. Mothers who had immediate postnatal care were 27% and mothers who had good knowledge about ENC were 36% more likely practice ENC. Mothers who faced complications during delivery were 80% less likely and with wealth index in the 2nd quintile were 74% more likely to practice ENC.

The prevalence of good ENC assesses in this study is in line with research conducted in North West Ethiopia (40.6%), higher than studies conducted in Awabel District, Amhara region of Ethiopia (23.1%) and Eastern Uganda (11.7%) and lower than two studies done in South West Ethiopia (59.5%) and Eastern Tigray of Ethiopia (92.9%) [12–14, 20, 21]. Health system-related factors contributing to this difference could be due to the extensive work of health extension workers and various health care institutions in awareness creation on ENC in the study area. Socio-cultural factors might include differences in the socio-cultural composition of the study groups, while other factors might include differences in the study period (e.g. studies conducted during harvest season may have differing results, and thus should and cannot be compared) and methodological weakness of the studies.

In this study 39.8% of mothers responded that substances were applied on the stump; this finding was supplemented by qualitative findings. This particular finding is higher than that found in four regions of Ethiopia (Amhara, Oromia, SNNPR and Tigray) at 21%. But, lower than the study conducted in Eastern Tigray of Ethiopia (56.8%) on another hand the qualitative finding was congruent with study done in Oromia Region of Ethiopia[13, 20, 22, 23]. The reason for the above difference may be due to socio-cultural factors. The majority (95.4%) of study participants stated that newborns were wrapped by new cloth before placenta expulsion immediately after delivery. This is higher as compared with study conducted in North West Ethiopia which was 57.5%[12]. The reason for this could be due to mothers’ better awareness about thermal care by extensive work of health extension workers in community-based newborn programs and home to home visits.

As shown by this study 59.8% of mothers stated that breastfeeding was started within one hour after delivery. This is low as compared to similar studies done in Eastern Tigray of Ethiopia and North West Ethiopia which were 93.2% and 62.1% respectively. However, this is relatively high when compared to study conducted in Awabel District, Amhara region of Ethiopia which was 41.6% [12, 13, 20]. This difference may be due to socio-demographic factors, awareness creation gap on breastfeeding initition time and socio-cultural factors. The majority (90.2%) of study participants stated colostrum was given for the newborn. This was congruent with the pervious study done in Ethiopia which was 98.3%[20]. The qualitative finding of this study also supplements the quantitative finding. However, it contradicts with study done in Oromia Region of Ethiopia reported that colostrum or first milk was not given for the newborn because it causes infection to the newborn[23]. The reason for this may be due to socio-cultural difference.

Regarding bathing time, 76.2% of study participants stated that their newborns were bathed after 24 hours. This result is congruent with similar study done in Eastern Tigray of Ethiopia (78.4%) and high as compared to two studies done in North West Ethiopia (60.8%) and Awabel District, Amhara Region of Ethiopia (34.4%) [12, 13, 20]. This may be due to the extensive work of health extension workers on a home to home basis. The qualitative finding is congruent with studies done in South West of Ethiopia and Oromia Region of Ethiopia stated that newborns were washed by warm water because they consider as dirty or contaminated by blood [14, 24]. The reason for is may be lack of knowledge about thermal care and socio-cultural factors.

In this study, mothers with a wealth index in the 2^nd^ quintile were 1.74 times more likely to practice good ENC. This is in line with a study conducted in Eastern Uganda[21]. Mothers who had sought antenatal care were 3.13 times more likely to practice good ENC. This is congruent with studies done in Northern Ghana, Eastern Uganda, Sindhuli District of Nepal, North West Ethiopia [12, 18, 21, 25]. The odds of practice were 2.90 among mothers who attended pregnant mothers meeting. This is in line with study done in Awabel District, Amhara Region of Ethiopia[13]. Mothers who had immediate postnatal care were 3.27 times and good knowledge about essential newborn care was 7.36 times more likely to practice essential newborn care. This is inline with studies done in Awabel District, Amhara Region of Ethiopia, Nepal, Eastern Tigray of Ethiopia, and Bachauli and Khairahani [13, 20, 25, 26]. The reason for this is mothers who had PNC discussed and were counseled about essentail newborn care with health extension workers or other health care providers and mothers who had good knowledge about essential newborn care is more likely to practice essential newborn care. In this study mothers who faced complication during delivery were 80% less likely to practice essential newborn care. The reason for this is it evident that mothers in complicated situation not gave care for their newborns unless conditions were resolved.

This contradicts with studies conducted in North West Ethiopia, South West Ethiopia, Nepal, Eastern Ghana and Eastern Tigray of Ethiopia [12, 14, 18, 20, 25]. The reason for this difference is due to the fact that health extension workers and other health care providers create awareness about practice for all mothers at home base regardless of age, educational level and occupation. In this study, mothers who were assisted by a skilled birth attendant during delivery were not significantly associated with good ENC practice. This is incongruent with a study done in Eastern Uganda[21]. The reason for this difference may be due to the case that some mothers reside in traditional mal-practices even if the delivery care was given by skilled birth attendant either at health care institution or at home which put the newborn in ill health.

The limitation of this study was respondents might be subjected to recall bias because the mothers failed to remember what they did for their infant in the early neonatal period. The study might not show a cause and effect relationship because the study design was cross-sectional.

## Conclusions

This study indicated that the level of essential newborn care practice was low. There are mal-practices in which 39.8% applied substances on the stump, 14.3% and 12.1% of the study participants used un-boiled instruments to cut the cord and given pre-lacteal feeding for their newborns immediate after delivery respectively.

In general, this study identified that antenatal care, attending pregnant mothers meeting, immediate postnatal care, wealth index in the 2^nd^ quintile and good knowledge about ENC were independent positive predictors for good ENC practice, whereas faced complication during delivery was negative predictor for good ENC practice.

As indicated by this study, age of mother, educational status and occupation of the mother were not significantly associated with good ENC practice. This key finding has important implications for the training of ENC care providers and health extension workers who are tasked with improving ENC delivery and uptake. For example, given that educational status and occupation of the mother are not significant predictors of good ENC, it is clear that community-based strategies are effective knowledge dissemination measures.

However, given the prevalence of malpractice, there may be opportunities to engage in conversations with prominent community leaders who may be influential in altering these practices and ideally, promote the uptake of good ENC practices.

Additionally, strengthen awareness creation activities on ENC through disseminating health information and developing communication strategies to promote positive behaviours both at facility and community level. Health facilities should regularly provide ENC for newborns and take opportunities to counsel the mothers about ENC during pregnant mothers meeting and MCH services sessions. This research has provided a sound basis for the improvement of ENC information dissemination and uptake, ideally resulting in improved practices. Further research should assess the degree to which interventions designed to maximize ENC are effective.

## Supporting information

S1 Tool This is the S1 English and Gammogna version tool. (DOCX)
