# Supplementary material for: Essential newborn care practice and its predictors among mother who delivered within the past six months in Chencha District, Southern Ethiopia, 2017
Source: PLoS One. 2018 Dec 11;13(12):e0208984. doi: 10.1371/journal.pone.0208984 (PMC6289501; doi:10.1371/journal.pone.0208984)
Supplement: S1 Tool — (DOCX) [file pone.0208984.s001.docx]

**English Version Participant Information Sheet and Voluntary Consent Form**

My name is (name of the data collector/interviewer). I am working as a data collector for the study being conducted in this community by Abera Mersha Mamo who is studying for his Master’s degree at Haramaya University, the College of Health and Medical Sciences. I kindly request you to lend me your attention to explain you about the study and being selected as the study participant.

**The study/project title:** “Essential newborn care practice and its predictors among mother who delivered within the past six months in Chencha District, Southern Ethiopia, 2017.”

**Purpose/aim of the study:**

The findings of this study will give paramount importance for Chencha District health office, different non-governmental organization and policy makers to plan intervention programs and to create awareness about the consequences about not giving effective essential newborn care for the baby. It creates awareness for health care institution about factors that make community not using health care institution and why do you not apply essential newborn care practices in a way which was recommended by health extension workers and other health care providers. Moreover, the aim of this study is to write a thesis as a partial requirement for the fulfillment of a Master’s Program in Maternity and Neonatal Nursing for the principal investigator.

**Procedure and duration**

If you are willing to participate in this study, you need to understand and put the signature on the agreement form. Then after, I will be interviewing you using a questionnaire to provide me with pertinent data that is helpful for the study. There are 88 questions to answer where I will fill the questionnaire by interviewing you. The interview will take about a maximum of 50 minutes, so I kindly request you to spare me this time for the interview.

**Risk/ Discomfort and Benefits**

There is minimal risk in participating in this research project. But, you are required to willingly spare time to respond to interviewed question from data collector.

If you participate in this research project, there may not be direct benefit to you but your participation is likely to help us in essential newborn care practice. Ultimately, this will help us to identify the gap and take the appropriate intervention by the authorized stakeholder.

**Confidentiality**

The information collected from this research project will be kept confidential and information about you that will be collected by this study will be stored in a file, without your name, but a code number assigned to it. In addition, it will not be revealed to anyone except the principal investigator and will be kept locked with key.

**Right to refuse or withdraw**

You have full right to refuse from participating in this research. You can choose not to respond to some or all questions if you do not want to give your response. You have also the full right to withdraw from this study at any time you wish, without losing any of your right.

**Contact Address**

If there are any questions or enquires any time about the study or the procedures, please contact:

Abera Mersha Mamo

*Cell Phone: +251910389538* ***OR*** *+251961413332*

*E-mail:* [*mershaabera@gmail.com*](mailto:mershaabera@gmail.com) ***OR*** [*mersha.abera@yahoo.com*](mailto:mersha.abera@yahoo.com)

Contact address of the responsible Institutional Health Research Ethics Review Committee (IHRERC) at office phone 0254660708 or P.O. Box 235, Harar.

**House Code**

**Declaration of Informed Voluntary Consent**

I have read/ she/ He were read for me the participant information sheet. I have clearly understood the purpose of the research, the procedures, the risks and benefits, issues of confidentiality, the rights of participating and the contact address for any queries. I have been given the opportunity to ask questions for things that may have been unclear. I was informed that I have the right to withdraw from the study at any time or not to answer any question that I do not want. Therefore, I declare my voluntary consent to participate in this study with my initials (signature).

Name and Signature of Participant: _______________________________________Date_______________Time_____________

Name and Signature of Data Collector: _______________________________________Date____________Time ____________

***Thank you!!!***

***……………………………………………………………………………………………………………………………………………………………………………………………………………………………………………………………………………………***

**Declaration of Informed Voluntary Consent**

I have read/ she/ He were read for me the participant information sheet. I have clearly understood the purpose of the research, the procedures, the risks and benefits, issues of confidentiality, the rights of participating and the contact address for any queries. I have been given the opportunity to ask questions for things that may have been unclear. I was informed that I have the right to withdraw from the study at any time or not to answer any question that I do not want. Therefore, I declare my voluntary consent to participate in this study with my initials (signature).

Name and Signature of Participant: _______________________________________Date_______________Time_____________

Name and Signature of Data Collector: _______________________________________Date____________Time ____________

***Thank you!!!***

**English Version Questionnaire**

**HARAMAYA UNIVERSITY**

**SCHOOL OF GRADUATE STUDIES**

**Dear Respondents**

This questionnaire is prepared to assess the essential newborn care practice and its predictors among mothers who delivered within the past six months in Chencha District, Southern Ethiopia, 2017. The assessment is made for the partial fulfillment of MSc Degree in Maternity and Neonatal Nursing. The questionnaire contains both closed and open ended questions and will be interviewed. You are therefore kindly requested to provide genuine response to the questions. The information you provide is confidential and is used only for the purpose of this study. If you have any question, don‘t hesitate to ask the data collector.

Your cooperation and participation until the completion of the interview is very necessary for the successful completion of the assessment.

***Thank you in advance for your cooperation!!!***

Data collectors sign: _____________

**Interview Record for Quantitative Data Identification Number**

Questionnaire developed for essential newborn care practice and its predictors among mothers who delivered within the past six months in Chencha District, Southern Ethiopia, 2017

| **S.No.** | **Question** | **Response** | **Skip** |
| --- | --- | --- | --- |
| **Part I: Socio- demographic Characteristics** | | | |
| 101 | Age of the mother | /_____________/ in complete year |  |
| 102 | Marital status | 1. Married 2. Divorced 3. Widowed 4. Single 5. Separated |  |
| 103 | Educational status of mother | 1. Cannot able to read and write 2. Can read and write 3. Grade 1-8 4. Grade 9-12 5. College and above |  |
| 104 | Religion | 1. Orthodox 2. Catholic 3. Protestant 4. Muslim 5. Traditional |  |
| 105 | Occupation of mother | 1. House wife 2. Merchant 3. Government employer 4. Daily laborer 5. Other, Specify___________________________ |  |
| 106 | Occupation of husband | 1. Farmer 2. Merchant 3. Government employer 4. Daily laborer 5. Other, specify___________________________ |  |
| 107 | Place of residence | 1. Urban 2. Rural |  |
| **Part II: Socio- economic status/ Wealth index questions** | | | |
| 201 | Does any member of this household has own any agricultural land? | 1. Yes 2. No | If “No” skip to Q205 |
| 202 | How much (local units) of agricultural land do members of this household own? | ________________________________( in Hectare) |  |
| 203 | Is the land cultivated? | 1. Yes 2. No | If “No” skip to Q205 |
| 204 | How much land is cultivated (local unit)? | ________________________________(in Hectare) |  |
| 205 | Does your family have any stored grains/cereals in the house? | 1. Yes 2. No | If “No” skip to Q207 |
| 206 | How many “kunital”? | ________________________________(in number) |  |
| 207 | Does this household own any livestock, herds, other farm animals, or poultry? | 1. Yes 2. No | If “No” skip to Q209 |
| 208 | How many of the following animals, does this household own? | \| **Animals** \| **Number** \| \| --- \| --- \| \| Milk cows, oxen or bulls \|  \| \| Horses, donkeys, or mules \|  \| \| Goats \|  \| \| Sheep \|  \| \| Chicken \|  \| \| Beehives \|  \| |  |
| 209 | Which of the following does your household have? *(Record observation)*  (*Multiple answers possible)* | 1. Electricity 2. Watch 3. Radio 4. Television 5. Mobile Telephone 6. Non Mobile Telephone 7. Chair 8. Table 9. Bed 10. Electric Mitad 11. Other (specify)__________________________ |  |
| 210 | What is main current source of drinking water for members of your house hold? | 1. Piped water 2. Public Tap/Stand Pipe 3. Borehole 4. Protected well 5. Unprotected well 6. Protected Spring 7. Unprotected Spring 8. River/Ponds/Stream/Dam 9. Other (specify) __________________________ |  |
| 211 | Do you do anything to the drinking water to make it safer to drink? | 1. Yes 2. No |  |
| 212 | What is the main source of water used by your household for other purposes such as cooking and hand washing? | 1. Piped water 2. Public Tap/Stand Pipe 3. Borehole 4. Protected well 5. Unprotected well 6. Protected Spring 7. Unprotected Spring 8. River/Ponds/Stream/Dam 9. Other (specify)__________________________ |  |
| 213 | What kind of toilet facility do members of your household usually use?  *(Record Observation)* | 1. Flush to piped sewer system 2. Flush to septic tank 3. Pit latrine with slab 4. Pit latrine without slab 5. Ventilated improved pit latrine 6. No facility/bush/field 7. Other (specify)__________________________ |  |
| 214 | What are the main materials of the floor of house?  *(Record observation)* | 1. Earth/Sand 2. Wood planks 3. Palm/Bamboo 4. Ceramic Tiles 5. Cement 6. Other (specify)__________________________ |  |
| 215 | What are the main materials of the roof of house?  *(Record observation)* | 1. Thatch/straw 2. Leaf/Earth/ Mud/Cow dung 3. Wood planks, cardboard 4. Finished roof (iron, tin, finished wood, cement, ceramic) 5. Other (specify)__________________________ |  |
| 216 | What is main material of the exterior walls of house?  *(Record observation)* | 1. Simple wall with mud or local materials 2. Bamboo or stone with mud, plywood, cardboard 3. Finished walls; cement, brick, stone with cement, wood planks 4. No outside walls 5. Others (specify)_________________________ |  |
| 217 | How many members are live in your household? | _____________________________(in number) |  |
| 218 | How many rooms are there for your house? | _____________________________(in number) |  |
| 219 | Do you have a separate room which is used as a kitchen?  *(Record observation)* | 1. Yes 2. No |  |
| 220 | What type of fuel do you mainly use for cooking?  (*Multiple answer possible)* | 1. Electricity 2. Wood 3. Kerosene 4. Animal dung 5. Charcoal 6. Shrubs/Grass 7. Agricultural crops 8. Other (specify)__________________________ |  |
| 221 | Which means of transport (vehicles) does any member of your household have? | 1. Bicycle 2. Motor cycle 3. Animal-drawn cart 4. Car/truck 5. None |  |
| 222 | Does any member of this household have a bank or micro-finance saving account? | 1. Yes 2. No | If “No” skip to Q301 |
| 223 | How much of money? | __________________________(Ethiopian Birr) |  |
| **Part III: Maternal and Child Health Services** | | | |
| 301 | Do you have Ante Natal Care visit? | 1. Yes 2. No | If “No” Skip to Q 306 |
| 302 | How many times you received Ante Natal Care service from health care provider? | ___________________________(in number) |  |
| 303 | Did you receive health information during your Ante Natal Care? | 1. Yes 2. No | If “No” Skip to Q 306 |
| 304 | If above answer is “Yes” lists them? | ___________________________________________ |  |
| 305 | Have you advised about essential newborn care practice during Ante Natal Care visits? | 1. Yes 2. No |  |
| 306 | Have you attended monthly pregnant mother’s group meeting? | 1. Yes 2. No |  |
| 307 | Where you gave birth? | - - - 1. Health center       2. Hospital       3. Health post       4. Home |  |
| 308 | Do you get assistance during delivery from skilled birth attendant? | 1. Yes 2. No | If “No” Skip to Q 310 |
| 309 | Who gave delivery assistance for you? | 1. Health care provider 2. Family 3. Neighbor 4. Relatives(mother in law) 5. Traditional Birth Attendants (TBAs) 6. Health Extension Workers (HEWs) 7. Other, Specify___________________________ |  |
| 310 | What was your mode of delivery? | - - - 1. Spontaneous vaginal delivery       2. Instrumental assisted delivery       3. Caesarean section | For institutional delivery only |
| 311 | Had you faced any type of complication during the delivery? | 1. Yes 2. No | If “No” Skip to Q 313 |
| 312 | If the above answer is “Yes” lists them? | ___________________________________________ |  |
| 313 | Who gave care for you after delivery? | 1. Health Extension Worker (HEW) 2. Family/ mother in law 3. Neighbor 4. My mom 5. Other, Specify___________________________ |  |
| 314 | Do you have immediate Post Natal Care visit? | 1. Yes 2. No | If “No” Skip to Q 316 |
| 315 | Have you advised about neonatal danger signs during Post Natal Care visits? | 1. Yes 2. No |  |
| 316 | Does your child is immunized? | 1. Yes 2. No | If “No” Skip to Q 318 |
| 317 | If the above answer is “Yes” states them? | __________________________________________ |  |
| 318 | Where you gave birth for index child? | 1. My home 2. Health Center 3. Hospital 4. In mom home |  |
| 319 | How many times you gave birth? | _______________________________________ |  |
| **Part IV: Source of Information about Essential Newborn Care practices and Health Workers Counseling related question** | | | |
| 401 | Have you ever heard/ counseled about Essential Newborn Care practice? | 1. Yes 2. No | If “No” skip to Q 404 |
| 402 | If above answer is “Yes”, about What? | - - - 1. About how to use common house delivery kit       2. About when to initiate breastfeeding       3. About when to bath the newborn |  |
| 403 | From which source you heard the information? | 1. Health professionals 2. Health Extension Workers (HEWs) 3. Health Development Army (HDA) 4. Neighbors 5. Friends 6. Media 7. Other, Specify___________________________ |  |
| 404 | Have you ever heard about neonatal danger signs | - - - 1. Yes       2. No | If “No” skip to Q 501 |
| 405 | From which source you heard the information? | 1. Health Professionals 2. Health Extension Workers (HEWs) 3. Health Development Army (HDA) 4. Neighbors 5. Friends 6. Media 7. Other, Specify___________________________ |  |
| **Part V: Birth Preparedness** | | | |
| 501 | During your last pregnancy did you make any preparations for your delivery? | 1. Yes 2. No | If “No” skip to Q601 |
| 502 | What preparations did you make for the delivery? | 1. Financial 2. Transport 3. Food 4. Identification of skilled birth attendant 5. Identification of facility 6. Blood 7. Clean delivery kit 8. Clothes for new born 9. Other, Specify___________________________ |  |
| 503 | During your last pregnancy did you arrange for a place to deliver your child? | 1. Yes 2. No | If “No” skip to Q505 |
| 504 | Where did you arrange to deliver your child? | 1. Hospital 2. Health center 3. Health post 4. Other, Specify___________________________ |  |
| 505 | Have you counseled about preparation for breast feeding | 1. Yes 2. No |  |
| 506 | Have you counseled on child feeding options? | 1. Yes 2. No |  |
| **Part VI: Knowledge related questions** | | | |
| 601 | Which instrument is used to cut the cord? | 1. New blade 2. Used blade 3. Knife 4. Scissor 5. Do not know |  |
| 602 | Which material is used to tie the cord? | 1. String or thread 2. Cord tie 3. Cord clamp 4. Do not know |  |
| 603 | At which time after delivery breastfeeding is initiated? | 1. Immediately after delivery 2. Within one hour after delivery 3. After one hour of delivery 4. After placenta is removed 5. Do not know |  |
| 604 | At which time after delivery bathing of newborn is recommended? | 1. Immediately after delivery 2. After 24 hour of delivery 3. Before 24 hour of delivery 4. Do not know |  |
| 605 | Do you know any neonatal danger signs? | 1. Yes 2. No |  |
| 606 | How many neonatal danger signs do you now? | _____________________________(in number) |  |
| 607 | Mention neonatal danger signs?  *(Multiple response is possible or ask us Yes/No question)* | - - - 1. Poor sucking or not able to breastfeeding       2. Fever  1. Fast breathing 2. Difficulty of breathing 3. Lethargic or unconscious 4. Hypothermia 5. Convulsion 6. Umbilical infection/such as redness of the cord 7. Yellowish discoloration of palms/soles 8. Vomiting |  |
| **Part VII: Practice related questions** | | | |
| 701 | Which instrument was used to cut the cord? | - - - 1. New blade       2. Used blade       3. Knife       4. Scissor       5. Other, Specify____________________________ |  |
| 702 | Was the instrument used to cut cord boiled prior to use? | Yes  No |  |
| 703 | Which material was used to tie the cord? | 1. String or thread 2. Cord tie 3. Cord clamp 4. Other, Specify____________________________ |  |
| 704 | Did anybody apply anything on the stump after the baby’s cord was cut? | 1. Yes 2. No | If “No” skip to Q706 |
| 705 | What did he/she apply? | 1. Ointment/powder 2. Butter 3. Animal dung 4. Ash 5. Other(specify)___________________________ |  |
| 706 | Is eye care given for the newborn? | 1. Yes 2. No | If “No” skip to Q708 |
| 707 | If the above answer is “Yes” by whom? | ___________________________________________ |  |
| 708 | How long after birth was your baby bathed for the first time? | 1. Immediately after delivery 2. After 24 hour of delivery 3. Before 24 hour of delivery |  |
| 709 | Temperature of the water is? | 1. Warm 2. Cold |  |
| 710 | Was your baby wiped off/dried within ten minute? | 1. Yes 2. No |  |
| 711 | Was your baby wrapped in cloth or put on mother's body and covered with cloth before the placenta was delivered? | 1. Yes 2. No | If “No” skip to 713 |
| 712 | Which types of cloth you used to wrap the baby after bath? | 1. New cloth 2. Clean and dry old cloth 3. Soiled and old cloth 4. Other, Specify___________________________ |  |
| 713 | How long after birth did you first put the baby on the breast? | 1. Immediately after delivery 2. Within one hour after delivery   After one hour of delivery |  |
| 714 | Did you give the first liquid (colostrum) that came out from your breasts? | 1. Yes 2. No | If “Yes” skip to 716 |
| 715 | What did you do with the first liquid (colostrum)? | ___________________________________________ |  |
| 716 | Did you give anything to drink other than breast milk in the first three days after birth? | 1. Yes 2. No | If “No” skip to 801 |
| 717 | If the above answer is “Yes”, which fluid you gave? | 1. Plain water 2. Butter 3. Animal Milk 4. Honey 5. Other, Specify___________________________ |  |
| **Part VIII: Health Care System related questions** | | | |
| 801 | Which health institution is found in your surrounding? | 1. Hospital 2. Health center 3. Health Post 4. Other, Specify___________________________ |  |
| 802 | Is those institutions provide Essential Newborn Care and create awareness in the community? | 1. Yes 2. No |  |
| 803 | The health care provider in the institution who give services related to Essential Newborn Care | 1. Health Extension Workers and Health Development Army 2. Nurses 3. Midwives 4. Doctors 5. Public Health officers 6. Others, Specify___________________________ |  |
| 804 | Distance to health institution from your home? | __________________________(Km) |  |

***Many Thanks!!!***

## English Version Qualitative Guide

**HARAMAYA UNIVERSITY**

**POST GRADUATE PROGRAM DIRECTORATE**

**Dear participant**

This qualitative guide for focused group discussion is prepared to assess the essential newborn care practices and its predictors among mothers who delivered within the past six months in Chencha District, Southern Ethiopia, 2017. The assessment is made for the partial fulfillment of MSc Degree in Maternity and Neonatal Nursing. The guide contains only open ended questions and will be discussed with your group. So, your group members are therefore kindly requested to discuss and provide genuine response to the questions. The information you provide is confidential and is used only for the purpose of this study. If you have any question, don‘t hesitate to ask the data collector.

Your cooperation and participation until the completion of the discussion is very necessary for the successful completion of the assessment.

***Thank you in advance for your cooperation!!!***

Data collectors sign: _____________

**A Guide for qualitative part of the study which is supportive for quantitative part (focused group discussion guide)**

1. How you give essential newborn care for newborn?
2. What are different cultural/traditional practices/belief related to essential newborn care regarding the three domains (cord care, thermal care and breastfeeding after delivery)
3. Do you have any awareness/information regarding essential newborn care from different groups in your surrounding? What are those information’s?

**Gammogna Version Participant Information Sheet and Voluntary Consent Form**

Ta sunthay (oyisha oyichiza danna suntha). Tanni Abera Mersha Mamoyi nam77atho digire othanas koshiza piligethas maraja shishiza gooda. Haramaya yuniverisitten, xennane hakkime kollejen ayotta hannotane 0-28 gallasa gakanaw de77iza pusha nayita hannota porogirame. Heyisa gishaw kalidi de77ezza ubba piligetha honnata siyanassine wozzana imidi kalannas daroppe bonichada yotta77es.

**Xinnate mega qoffay:** “Pusha nayiti yeletishin immetiza maadonne ennita hannota ubba politonita malla othiza metota ussupun aginna giddon yelida ayotan Cencha Woraddan, Duggeha Tophphiya, 2017 marotetha layithan.”

**Ha piligetha go77ay**

Ha piligethay daro go77ay de77es. Hessa gidopee wannati Cencha Woradda xenna xiffete keethi, mangiste keethappe hara maddo immizza dirijitetinne woggane halicho kesiza kifileti ubbay pusha nayiti yeletishin immetiza maado bolla kooshiza qoffisso immanas halicho kesanassine maaran immitonita gidiko gathiza metota bolla timirite immanas go77es. Qasseka Woraddan dizza xenna go77a immiza osso keethatinne qabalen qabelen dizza xenna ekisitenshine ossanchati ubbay ayotti pusha nayti yeletishen immetiza maddo maaran polonitta malla othiza hannotata piligi erannas daroppe maades.

Ubbappe adhdho, ha piligetha go77ay danna Abera Mersha Mamoy nam77atho digire ayottane 0-28 gallasa gakanew de77iza nayitas immetiza maddo bolla othanas koshiza gishaw.

**Piligetha hannota**

Ha piligethan gelannas dossadis, ubba xinnate/piligetha hannottay taasi geliddes gikko pirimma/malatta paramma. Hessappe guye tanni oyisha oyichishin zaro immasa. Ha piligethas 80 oyishay de77es. He oyisha la77anaw/wurisanaw 40 deqiqa kooshes. Heyisa gishaw oyisha la77ana/wurisana gakkanaw wozannani kalanna malla daroppe bonchada oddayis/yotayis.

**Ha piligetha metto gathonne go77a**

Ha piligethay, ha piligethan geliza ayotta bolla guutha woyikko daroppe qohonitta metto gathanaw danda77es. Giddoppe attin ennita dossan osso agiddi oyishay wuranna gakkanaw osso birishidi oyisha zarroy de77es.

Ha piligethan geliza ayottas kisse me77ey woyikko birra qanixoy denna. Giddoppe attin ennita zarroy ha piligetha polanawunaw daroppe maades. Qasseka pusha nayita maddo bolla de77eza metota piligi erenawunne he metota kalidi birishiza, kalidi xelizanne timirite immiza cugattasine osso kethatas daro go77a immes.

**Xurra naggo**

Immetidda zarroy woyikko qoffay ayinika oggen hara assi siyanawunne xaffetida xuffe/mega qoffa nababanaw danda77ena. Immetidda zarroy ubbay zarro immida ayotta sunthayinne harabba ayotta assi erennaw dandayizza marajay denna. Giddoppe attin “koddey” immetes. Qasseka zarro ubbay piligetha othiza dannappe hara assi xelanaw danda77ena. Hessape guye kuliffen kulifettidi woyikko nagettidi uttes.

**Piligethappe kezzannas/ Agannas**

Ha piligethan gelontta aganaw danda77ettes. Qasseka oyishottas ubbas woyikko issi issi oyishottas zarro immonitta aganaw danda77ettes. Ha piligethappe innite dossida saaten kezannaw “maffetey” de77es.

**Addirasha**

Oyishay woyikko qoffisoy dikko kalidi de77iza addirashan demannaw danda77etes.

Sunthay: Abera Mersha Mamo

Silike: 0910389538 **woyikko** 0961413332

*E-melle:* [*mershaabera@gmail.com*](mailto:mershaabera@gmail.com) ***woyikko*** [*mersha.abera@yahoo.com*](mailto:mersha.abera@yahoo.com)

Yuniverisitten xenna piligetha kalizza kommitte (“IHRERC”) addirasha: Birro Silike: 0254660708/ positta saxinee payiddo 235, Harere.

***Keetha Koode***

**Dossa Geeshi Yottiza Mega Qoffa**

Taani ha piligetha ubba hannota nababadis/taasi nababadis/nababadus. Taani ha piligetha go77ay, ubba hannottay, piligethay metto gathonitta aggoy, xurra naggoy, piligethappe dossida saaten kezannaw danda77etiza “mafiteyinne” oyishay woyikko qoffisoy dikko gaganna addirashay ubbay immetides. Gellonabay dikko oyichanna “iddiley” immetides. Ha piligethappe dossida saaten kezanawunne oyishattas ubbas woyikko issi issi taani zaranaw dossonita oyishatas zarro immontta aganaw taasi yotetides. Hessa gishaw ha piligethan taani gelidday dossan gididayissa kaladda garissan dizza bottan pirimman wothayis.

Ta sunthayinne pirimma__________________________________________________________

Gallasa____________________Saate____________

Oyisha oyichiza danna sunthayinne pirimma__________________________________________

Gallasa____________________Saate_____________

***Daroppe galatayis!!!***

***……………………………………………………………………………………………………………………………………………………………………………………………………………………………………………………………………………………***

**Dossa Geeshi Yottiza Mega Qoffa**

Taani ha piligetha ubba hannota nababadis/taasi nababadis/nababadus. Taani ha piligetha go77ay, ubba hannottay, piligethay metto gathonitta aggoy, xurra naggoy, piligethappe dossida saaten kezannaw danda77etiza “mafiteyinne” oyishay woyikko qoffisoy dikko gaganna addirashay ubbay immetides. Gellonabay dikko oyichanna “iddiley” immetides. Ha piligethappe dossida saaten kezanawunne oyishattas ubbas woyikko issi issi taani zaranaw dossonita oyishatas zarro immontta aganaw taasi yotetides. Hessa gishaw ha piligethan taani gelidday dossan gididayissa kaladda garissan dizza bottan pirimman wothayis.

Ta sunthayinne pirimma__________________________________________________________

Gallasa____________________Saate____________

Oyisha oyichiza danna sunthayinne pirimma__________________________________________

Gallasa____________________Saate_____________

***Daroppe galatayis!!!***

**HARAMAYA YUNIVERESITE**

**MIRREQAPPE GUYE TIMIRITE KEETHA**

**Oyisha Zarranayitto**

Ha oyishay gigetidday “Pusha nayiti yeletishin immetiza maadonne ennita hannota ubba politonita malla othiza metota ussupun aginna giddon sohon yelida ayotan Cencha Woraddan, Duggeha Tophphiya, 2017 marotetha layithan”. Ha piligethay othetizay nam77atho digire ayottane 0-28 gallasa gakkanaw de77iza nayitas immetiza maddo bolla othanas koshiza gishaw. Ha oyishay dorronne qallan zaretiza oyishata oyikides. Gidoppe attin oyishay oyisha oyichiza daanay qallan nababidi oyichishin zarroy immetes. Heyisa gishaw koshiza zarro immanas daroppe bonchada yota77es/ oyda77es. Nenni immida zarroy ubbay ha piligethasinne go77as kanche. Oyishay oyishetishen gelonnabay/qoffisoy dikko oyichannas guye gopa.

Ne maddotethayinne gelloy ha piligethasinne piligetha ubba polannas daroppe koshes.

***Daroppe galatayis!!!***

Oyisha oyichiza danna pirimma___________________

**Payiddon Patetizza Piligetha Oyisha Shakko Payiddo**

Oyishay gigetidday “Pusha nayiti yeletishin immetiza maadonne ennita hannota ubba politonita malla othiza metota ussupun aginna giddon sohon yelida ayotan Cencha Woraddan, Duggeha Tophphiya, 2017 marotetha layithan

| **O.py** | **Oyisha** | **Zarro** | **Dhiko** |
| --- | --- | --- | --- |
| **Basso I: Ayotaba Yottiza Hannota** | | | |
| 101 | Ayyi layitha/iddime | /_______/ kummetha layithan |  |
| 102 | Azzina gello hannota | - - - 1. Gelladus       2. Shagetadus       3. Azzinay hayqides       4. Gellabuku       5. Osso gishaw hara bottan de77es |  |
| 103 | Timirite tamarida hannota | 1. Nababonne xaffo errike 2. Nababonne xaffo erayas 3. 1-8 kifile tamaradis 4. 9-12 kifile tamaradis 5. Kollejenne kollejeppe adho |  |
| 104 | Ammano | 1. Orotodokisse 2. Missonne 3. Katolike 4. Issilamma 5. Bahile ammano |  |
| 105 | Ayyi osso | - - - 1. So ossanicha       2. Zalle77e       3. Kawwo ossanicha       4. Wolqqa osso       5. Harabba (yotta)________________________ |  |
| 106 | Awa osso | 1. Goshanicha 2. Zalle77e 3. Kawwo ossanicha 4. Wolqqa osso 5. Harabba (yotta)________________________ |  |
| 107 | Dizza soho | 1. Kattama 2. Gaxare |  |
| **Basso II: Dizza miisha woyikko durettethaba yottiza oyishata** | | | |
| 201 | Gaddey woyikko bittay dizze? | 1. Dees 2. Baawa | Zarroy “2” gidiko 205 py.oy.dhik. |
| 202 | Ayi kenna gadday woyikko bittay dizze? | ____________________________( Hekitaren) |  |
| 203 | Kaathi zeretidde? | 1. Zeretides 2. Zeretibenna | Zarroy “2” gidiko 205 py.oy.dhik. |
| 204 | Ayi kenna zeretiday? | _____________________________(Hekitaren) |  |
| 205 | Sohon shishetidda kaathi gootaran woyikko di77en dizze? | 1. Dees 2. Baawa | Zarroy “2” gidiko 207 py.oy.dhik. |
| 206 | Konittalen ayi kenna? | ____________________________(Payiddon) |  |
| 207 | Sohon so meehey dizze? | 1. Dees 2. Baawa | Zarroy “2” gidiko 209 py.oy.dhik. |
| 208 | Ayibbe dizzay? | \| **Meehe** \| **Payiddo** \| \| --- \| --- \| \| Maatha miza, Borra/Sanigga \|  \| \| Para/ Hare/ Baqqulo \|  \| \| Deesha \|  \| \| Doro/ Dorissa \|  \| \| Kutto \|  \| \| Matha \|  \| |  |
| 209 | Kalidi de77izayitappe awoyisatti sohon de77izo?  *(Xellada kunitha)*  *(Daro zaro immoy* *danda77ettes)* | 1. Mabiratte 2. Saate 3. Raddone 4. Televizhine 5. Mobayile silike 6. Soho silike 7. Utetha 8. Dharapheza 9. Aliga/ Esho 10. Mabiratte baashe 11. Harabba (yotta)________________________ |  |
| 210 | Usha haatha anappe go77eteti? | 1. Bonbba haathappe 2. Eqqidi goshetiza bonbbappe 3. Boketida qere holan shiqettida haathappe 4. Nagettidi diza hola haathappe 5. Nagottonita hola haathappe 6. Nagettidi diza pulto haathappe 7. Nagottonita pulto haathappe 8. Gogiza/shaafa/kanche/shiqettida haathappe 9. Harabba (yotta)________________________ |  |
| 211 | Usha haatha bolla dhale gujeza assi sohon dizze? | 1. Dees 2. Baawa |  |
| 212 | Ushappe hara, harabba othanas haatha anappe go77eteti? | 1. Bonbba haathappe 2. Eqqidi goshetiza bonbbappe 3. Bokettida qere holan shiqettida haathappe 4. Nagettidi diza hola haathappe 5. Nagottonita hola haathappe 6. Nagettidi diza pulto haathappe 7. Nagottonita pulto haathappe 8. Gogiza/shaafa/kanche/shiqettida haathappe 9. Harabba (yotta)________________________ |  |
| 213 | Ayi malla sheesha keetha go7etteti?  *(Xellada kunitha)* | 1. Haathi gujettiza tubbon biza 2. Haathi gujettiza tankeren shiiqiza 3. Kaamay diza bokettida hola 4. Kaamay donitta bokettida hola 5. Ayere kesiza tubboy diza 6. Sheesha keethay baawa/worran/ dembban 7. Harabba(yotta)_________________________ |  |
| 214 | Keetha wuggey ayiban ossetide?  *(Xellada kunitha)* | 1. Bitta/ Ancho/Uriqa/Tiffa meesha 2. Mitha phaliqetha 3. Woyisha phaliqetha/ qaarixa 4. Seramike 5. Siminitto meesha 6. Harabba(yotta)_________________________ |  |
| 215 | Keetha karroy/korinisey ayiban ossetide?  *(Xellada kunitha)* | 1. Maata/Buuxa 2. Qatta/ Uriqa/Tiffa meesha 3. Mitha phaliqetha/ Karittone 4. Wuridda karro (Qoriqoro, Wuridda mitha phaliqetha, Siminitto, Seramike) 5. Harabba (yotta)________________________ |  |
| 216 | Keetha eqqoy woyikko godday ayibin ossetide?  *(Xellada kunitha)* | 1. Uriqa mesha woyikko deeren dizza miisha 2. Woyisha woyikko uriqan oyketidda shucha, turran qashetidda, karittone 3. Wuridda eqqo/Godda; Siminitto, Xube, Siminitto oyketidda shucha, Mitha phaliqetha 4. Eqqoy baawa 5. Harabba (yotta)_________________________ |  |
| 217 | Appun assi sohon dizze? | _______________________________(Payiddon) |  |
| 218 | Keethas appun kifiley dizze? | _______________________________(Payiddon) |  |
| 219 | Shakettida kummay kathetiza keethay dizze?  *(Xellada kunitha)* | 1. Dees 2. Baawa |  |
| 220 | Qumma kathannas ayi go7etteti? | 1. Mabiratte 2. Mitha 3. Gaaze 4. Melidda tiffa 5. Kaasale 6. Maata/hunixare 7. Buuxa 8. Harabba (yotta)________________________ |  |
| 221 | Tiransiporttes go77iza miishatappe awoyisatti sohon dizza assasi dizzo? | 1. Bishikilite/Sayikile 2. Mootore bishikilite/Sayikile 3. Soho meehen goshetiza gaare 4. Maakina 5. Ayibika baawa |  |
| 222 | Banike keetha bukey/mayikirro fayinasse kuxebay dizze? | 1. Dees 2. Baawa | Zarroy “2” gidiko 301 py.oy.dhik. |
| 223 | Ayi kenna? | __________________________(Tophphiya Biiran) |  |
| **Basso III: Ayotta udethanne pusha/yidha nayita xenna kaletho hannota** | | | |
| 301 | Uddetha kaloy dizze? | - - - 1. Dees       2. Baawa | Zarroy “2” gidiko 306 py.oy.dhik. |
| 302 | Ayi kenna kaladda baddi? | ___________________________(Payiddon) |  |
| 303 | Uddetha kaladda xenna go77a zorre ekaddi? | 1. Ekaddis 2. Ekabeyike | Zarroy “2” gidiko 306 py.oy.dhik. |
| 304 | Bollan dizza zarroy “Ekaddis” gidiko ayibba ekaddi? | __________________________________________ |  |
| 305 | Uddetha kalishin pusha nayita yeletta maadonne zorrey immetide? | - - - 1. Immetides       2. Immetibena |  |
| 306 | Uddethi dizza ayotta zorren woyikko sikethan bettadi? | - - - 1. Bettadis       2. Bettabeyike |  |
| 307 | Anani yeladi? | 1. Xenna xabiyan 2. Hosppitalen 3. Xenna keelan 4. Sohon |  |
| 308 | Yeliso moyanichay/ hilanichay kalide/ madide? | - - - 1. Madides/kalides       2. Madibena/ Kalibena | Zarroy “2” gidiko 310 py.oy.dhik. |
| 309 | Yeliddappe guye maddo immeday onne? | 1. Xenna go77a immiza moyannicha 2. So assa 3. Shooro/Gutta 4. Dabbo(Azinna Ayo) 5. Gutta hilancha 6. Tenna ekisitenshine 7. Harabba(yotta)_________________________ |  |
| 310 | Ayiban yeladi? | 1. Taffe bankadda 2. Massaran maditada 3. Oppereshine othetada | Sohon yelidayitabicha oyicha |
| 311 | Yello bolla mettoy gakide? | - - - 1. Gakides       2. Gakibena | Zarroy “2” gidiko 313 py.oy.dhik. |
| 312 | Bollan dizza zarroy “Gakides” gidiko ayibe? | __________________________________________ |  |
| 313 | Yeliddappe guye maddo immeday onne? | 1. Xenna ekisitenshine 2. Soho assa/ Azzina ayyo 3. Gutta assa 4. Ta ayyo 5. Harabba(yotta)_________________________ |  |
| 314 | Yelethappe guye hannota kaloy dizze? | 1. Dees 2. Baawa | Zarroy “2” gidiko 316 py.oy.dhik. |
| 315 | Hannota kalishin nayita bolla itta metto gathiza hannotatan zorrey immetide? | 1. Immetides 2. Immetibena |  |
| 316 | Pushay kittibate katebettide? | 1. Katabetides 2. Katabetibena | Zarroy “2” gidiko 318 py.oy.dhik. |
| 317 | Bollan dizza zarroy “Katabetides” gidiko ayibba? | __________________________________________ |  |
| 318 | Sinitha pusha annan yeladdi? | 1. Ta sohon 2. Xenna xabiyan 3. Hospitallen 4. Ta ayye sohon |  |
| 319 | Appun yeladi? | _________________________(payiddon) |  |
| **Basso IV: Pusha nayiti yelethan immetiza maddo hannota zorre pulttonne xenna hilancha zorre immo hannota oyishata** | | | |
| 401 | Pusha nayiti yelethan immetiza maddo hannota bolla zorre siyaddi? | 1. Siyaddis 2. Siyabe77ike | Zarroy “2” gidiko 404 py.oy.dhik. |
| 402 | Bollan dizza oyishas zarroy “Siyaddis” ayi hannota? | 1. Soon diza mishata shishidi lo77o oggen yello hannota peshon 2. Dhanitha dhanitho ayide dommas koshiza hannota 3. Nayi yeletidappe guye ayide mecho koshiza hannota |  |
| 403 | Ayi pulittoppe zorre siyaddi? | 1. Xenna moyachatappe 2. Tenna ekesteshineppe 3. Dere maadiza ossanchatappe 4. Gutta assappe 5. Lageppe 6. Middiyappe 7. Harabba(yotta)_________________________ |  |
| 404 | Qere nayita bolla itta metto gathiza hannotata zorre siyaddi? | - - - 1. Siyaddis       2. Siyabe77ike | Zarroy “2” gidiko 501 py.oy.dhik. |
| 405 | Ayi pulittoppe siyaddi?? | 1. Xenna moyanichappe 2. Tenna ekesteshineppe 3. Dere maadiza ossanchatappe 4. Guttappe 5. Lageppe 6. Middiyappe 7. Harabba(yotta)_________________________ |  |
| **Basso V: Yellos gigitto hannota** | | | |
| 501 | Adhida uddethas yellos gigetaddi? | - - - 1. Gigetaddis       2. Gigetabe77ike | Zarroy “2” gidiko 601 py.oy.dhik. |
| 502 | Ayiban gigetaddi? | 1. Biiran 2. Ogge bussa hannotan/tiransportten 3. Koshiza qumman 4. Koshiza hilancha shakkidi erron 5. Koshiza xenna kalizza keetha shakkon 6. Koshiza suutha wothon 7. Yellos koshiza miishata shishon 8. Na77as koshiza ma77on 9. Harabba(yotta)_________________________ |  |
| 503 | Adhida uddethas yeliso go77a immiza keetha shaako bolla gigetaddi? | 1. Gigetaddis 2. Gigetabeyikke | Zarroy “2” gidiko 505 py.oy.dhik. |
| 504 | Ayi keetha bannas gigetaddi? | 1. Hosippitale 2. Xenna xabiya 3. Xenna keela 4. Harabba(yotta)_________________________ |  |
| 505 | Dhanitha dhanithannas gigitto bolla zorre ekaddi? | 1. Ekaddis 2. Ekabeyike |  |
| 506 | Pushas/ yidhas qumma immo doorotta bolla zorre ekaddi? | 1. Ekaddis 2. Ekabeyike |  |
| **Basso VI: Erro woyikko uqqata oyishata** | | | |
| 601 | Gul77a kanxannaw ayi miisha koshi? | 1. Oratha milace/ shigire 2. Go77ettida milace 3. Maasha 4. Maqase 5. Errike |  |
| 602 | Gul77a kanxidappe guye qachannas ayi koshi? | 1. Kirre/ shaya 2. Gul77a kachiza hakime keetha kirre 3. Gul77a xunniti oyikiza qaphe 4. Errike |  |
| 603 | Ayi saaten na77as dhanitha immo koshize? | 1. Yeletidappe guye esson 2. Yeletidappe guye issi saate giddon 3. Issi saateppe guye 4. Yiicoy wodhidappe guye 5. Errike |  |
| 604 | Na77ay yeletidappe guye appun saate giddon boola mecannaw koshize? | 1. Yeletidappe guye esson 2. Yeletidappe nam77i tammane oyiddi saateppe guye 3. Nam77i tammane oyiddi saateppe sinthan 4. Errike |  |
| 605 | 0-28 gallasa gakanaw dizza ya77ita bolla betiza itta metto gathiza hannota erray? | - - - 1. Erayis       2. Errike |  |
| 606 | Appun hannota erray? | _______________________________(payiddon) |  |
| 607 | Erizayita yotta?  *(Daro zaro immoy danda77ettes)* | 1. Dhanitha dhamontta aggo 2. Bolla misha 3. Esso esson shenippo 4. Shenippanaw wayisso/woliqatho 5. Labanno 6. Bollay daroppe erixo 7. Daroppe kokoro/ goffonitto kesso 8. Gul77ay madunixo woyikko zo77oto 9. Kushey woyikko tohoy qa77ay bichatto 10. Coosho |  |
| **Basso VII: Othiddi besso woyikko othon peesho oyishatta** | | | |
| 701 | Na77ay yeletishen gul77ay ayiban qanixetidde? | 1. Oratha milacen 2. Go77etida milacen 3. Maasha 4. Maqase 5. Harabba(yotta)_________________________ |  |
| 702 | Nenni go77etidda miishay misha haathan doyisetidde? | Doyisetides   - - - 1. Doyisetibenna |  |
| 703 | Gul77ay qanixetidappe guye ayiban qachetidde? | Kirren/ shayan  Gul77a qachiza hakkime keetha kirren  Gul77a xunniti oyikiza qaphen  Harabba(yotta)_________________________ |  |
| 704 | Gul77a bolla teyetiza miisha tiyidda assi dizze? | 1. Dees 2. Baawa | Zarroy “2” gidiko 706 py.oy.dhik. |
| 705 | Ayibbe tiyettiday? | 1. Qibatte/Dhile 2. Oyisa 3. Meehe tiffa/cooro/ osha 4. Buddo 5. Harabba(yotta)_________________________ |  |
| 706 | Ayiffe naggos koshiza dhaley immetide? | 1. Immetides 2. Immetibenna | Zarroy “2” gidiko 708 py.oy.dhik. |
| 707 | Zarroy Immetides gidiko, onne immeday? | __________________________________________ |  |
| 708 | Na77ay/yidhay yeletidappe guye appun saate giddon boolay mecettide? | 1. Yeletidappe guye esson 2. Yeletidappe nam77i tammane oyiddi saateppe guye 3. Nam77i tammane oyiddi saateppe sinthan |  |
| 709 | Boola mecannaw ayi haatha go77etideti? | 1. Pentida/ ho77eda haatha 2. Irixa haatha |  |
| 710 | Na77ay yeletidappe guye taamu deqiqa giddon boolay cariqan ucetidde/ bollimide? | 1. Bollimides 2. Bollimibenna |  |
| 711 | Na77ay yeletidappe guye yiicoy wodhana gakkanaw afilan kametide/ xaxetide? | 1. Xaxetides 2. Xaxetibena |  |
| 712 | Boolay mecetidappe guye ayi affilan xaxetide/ ma77ide? | 1. Oratha affilan/maayon 2. Mecetidda geesha affilan 3. Qita/ gali77a affilan 4. Harabba(yotta)_________________________ |  |
| 713 | Yeletidappe guye appun saate giddon dhanitho domadi/ dhanitha immadi? | 1. Yeletidappe guye esson 2. Yeletidappe guye issi saate giddon 3. Issi saateppe guye |  |
| 714 | Koyiro maatha na77as immadi? | 1. Immadis 2. Immabeyike | Zarroy “1” gidiko 716 py.oy.dhik. |
| 715 | Bollan dizza zarroy “Immabeyike” gidiko koyiro maatha wosittadi? | ________________________________________ |  |
| 716 | Dhanithappe hara miishay immetide? | 1. Immetides 2. Immetibenna | Zarroy “2” gidiko 801 py.oy.dhik. |
| 717 | Bollan dizza zarroy “Immetides” gidiko ayibe immetiday? | 1. Haatha 2. Oyisa 3. Mizza maatha 4. Essa 5. Harabba(yotta)_________________________ |  |
| **Basso VIII: Xenna go77ata immone go77eto hannota** | | | |
| 801 | Ukken ayi xenna go77a immiza keethay dizze? | 1. Hosippitale 2. Xenna xabiya 3. Xenna keela 4. Harabba(yotta)_________________________ |  |
| 802 | He xenna go77a immiza keethati pusha nayita maddo hannota bolla go77a immizzo? | 1. Immetes 2. Immetena |  |
| 803 | Go77a imizzayti onnatte? | 1. Tenna ekestenshinettane xenna go77a immiza kitanchata 2. Nerseta 3. Awalaje/ Yeliso Nerseta 4. Doketereta 5. Xenna mokenineta 6. Harabba(yotta)_________________________ |  |
| 804 | Sohoppe xenna go77a immiza keetha gakkanaw ayi kenna haaki? | ______________________(Km) |  |

***Daroppe galatayis!!!***

**Gammogna Version Qualitative Guide**

**HARAMAYA YUNIVERESITE**

**MIRREQAPPE GUYE TIMIRITE KEETHA POROGIRAME DAYEREKITORETE**

**Zorres Shiqidayitto**

Ha oyishay/mega qoffay zorretidi zarranas gigetidday “Pusha nayiti yeletishin immetiza maadonne ennita hannota ubba politonita malla othiza metota ussupun aginna giddon yelida ayotan Cencha Woraddan, Duggeha Tophphiya, 2017 marotetha layithan”. Ha piligethay othetizay nam77atho digire ayottane 0-28 gallasa gakanew de77iza nayitas immetiza maddo bolla othanas koshiza gishaw. Ha mega qoffay qallan zaretiza oyishata oyikides. Gidoppe attin oyishay oyisha oyichiza daanay qallan nababidappe woyikko yotidappe guye zorretiddi zarroy immetes. Hessa gishaw innite cuggay zorretidi koshiza zarro immanas daroppe bonchada yota77es/oyda77es. Innite immida zarroy/ zorrey ubbay ha piligethasinne go77as kanche. Zorrey dometidappe guye gelonabay/qoffisoy dikko oyichannas guye gopitte.

Innite maddotethaynne zorrey ha piligethasinne piligetha ubba polannas daroppe koshes.

***Daroppe galatayis!!!***

Oyisha oyichiza danna pirimma___________________

**Ha mega qoffay payiddon patetiza piligetha minthanas koshiza qallan patetiza piligethas maadiza zorres koshiza oyishata (“Gayide”)**

1. Pusha na77iti yeletishin wossiti maddo immeti?
2. Innite guttan woyikko sohon pusha na77iti yeletishin immetiza maddo immetiza woggati/ bahiley dikko hezzi sinththa mega qoffata (Gul77a boncho hannota, bolla moceppe naggo hannotane yeletidappe guye dhanitha immo hannota) bolla zorretite?
3. Hayisappe sinthan pusha na77iti yeletishin immetiza maddo hannota bolla innite siyadabay dizze? Ayiba siyideti?
